# COVID-19 and European carcerality: Do national prison policies converge when faced with a pandemic?

**DOI:** 10.1177/14624745211002011

**Published:** 2022-10

**Authors:** Olga Zeveleva, José Ignacio Nazif-Munoz

**Affiliations:** 3835 University of Helsinki, Finland; Université de Sherbrooke, Canada; Harvard University, USA

**Keywords:** carcerality, coronavirus, COVID-19, European prisons, penal nationalism, penal populism, prison policy, prisoners, public health

## Abstract

The article analyses an original dataset on policies adopted in 47 European countries between December 2019 and June 2020 to prevent coronavirus from spreading to prisons, applying event-history analysis. We answer two questions: 1) Do European countries adopt similar policies when tackling the COVID-19 pandemic in prisons? 2) What factors are associated with prison policy convergence or divergence? We analyze two policies we identified as common responses across prisons around the world: limitations on visitation rights for prisoners, and early releases of prisoners. We found that all states in our sample implemented bans on visits, showing policy convergence. Fewer countries (16) opted for early releases. Compared to the banning of visitation, early releases took longer to enact. We found that countries with prison overcrowding problems were quicker to release or pardon prisoners. When prisons were not overcrowded, countries with higher proportions of local nationals in their prisons were much faster to limit visits relative to prisons in which the foreign population was high. This research broadens our comparative understanding of European carcerality by moving the comparative line further East, taking into account multi-level governance of penality, and analyzing variables that emphasize the ‘society’ element of the ‘punishment and society’ nexus.

## Introduction

It is well established that prisons are particularly susceptible to infections, and people in prison face high risks of complications (Kinner et al., 2020; Montoya-Barthelemy et al., 2020; World Health Organization, 2020c). Moreover, socio-economically deprived groups and ethnic minorities are overrepresented in prisons, which implies that when COVID-19 enters prison walls, it will disproportionately affect those who are already vulnerable and marginalized (Ahmed et al., 2020; Beckett and Western, 2001; Ruddel, 2005; Todd-Kvam, 2018). In this context, we turn our attention to the ways in which national governments in the EU and in neighboring countries tried to prevent coronavirus from spreading to carceral settings. This research broadens our comparative understanding of punishment in Europe in three ways: first, we analyze a country sample that extends beyond the typical comparative penology focus on liberal democracies with longstanding capitalist histories (Brangan, 2020; Daems et al., 2013) by moving the comparative line further East (Haney, 2016; Lappi-Seppälä 2008: 314) to include postsocialist states like Belarus, Russia, Georgia, Kazakhstan. Second, we take into account multi-level governance of penality in the context of the involvement of international bodies in formulating COVID-19 policy recommendations, and European harmonization of prison policy (Piacentini and Katz, 2017; Van Zyl Smit and Snacken, 2009; Vaughan and Kilcommins, 2007). Third, we go beyond prison characteristics and analyze a broad range of variables that could be associated with prison policies, such as epidemic systems, political orientations of dominant parties, democracy, and GDP per capita. In this way, the country cases examined here represent vastly differing penal histories as well as uneven or divergent trajectories of harmonization with European policy, and the variables we analyze emphasize the ‘society’ element of the ‘punishment and society’ nexus. Our findings, then, speak to debates on the propensity of countries for welfare provision versus the propensity to incarcerate, discussed by, for instance, Wacquant (2009) and Sutton (2013); our attention to the variable of incarceration of foreigners also complicates the welfare versus incarceration dichotomy from the analytical angle of penal nationalism, as developed by Haney (2016) and Barker (2017, 2018).

It is premature to conduct cross-national comparative analyses of the effects of COVID-19 on prisons and related communities, as both data on infection and death rates in penal institutions are limited, and the threat of the pandemic is still present. Nonetheless, it is possible to begin empirical analyses of how prison policies designed to tackle COVID-19 have spread across the world. This paper, therefore, explores factors that may have led to prison systems in different countries to enact similar or differing policies at equivalent or varying speed. More specifically, this study examines how different countries have responded to the common threat of a COVID-19 outbreak in prisons, and how quickly they have done so. We analyse whether European countries converge in their prison policy responses to the pandemic regardless of national characteristics. Policy convergence is possible in light of the global nature of the pandemic, and against the backdrop of multi-level governance of prisons, as manifested in the common regulatory framework within the Council of Europe and calls from international organizations (including the World Health Organization, the United Nations Office on Drugs and Crime, the Office of the UN High Commissioner for Human Rights, and others) to address the heightened vulnerability of people in carceral settings. We analyse the type of policy adopted, as well as speed of adoption.

The article addresses the following research questions: First, do European countries adopt similar policies when tackling the COVID-19 pandemic in prisons? Second, what factors are associated with prison policy convergence or divergence? To do so, we analyze two policies that we identified, in our analysis of reports by NGOs, media, and prison services, as most common and comparable responses in different penal systems across the world (Zeveleva, 2020)^1^: first, limitations on visitation rights for prisoners; second, early releases or pardons of prisoners. These two policies can coexist within one country, and reveal the complexities and contradictions at work when policymakers try to prevent a pandemic from spreading to a prison system, or from a prison system. The first policy involves closing prisoners off from the outside world, which may represent a more punitive approach, as it takes a mental health toll on prisoners and makes it even more difficult to uphold relationships between prisoners and their families and friends (Fovet et al., 2020; Kothari et al., 2020; Montoya-Barthelemy et al., 2020). However, this policy could also be designed to protect prisoners from infections brought in from outside. On the other hand, a policy of release can be classified as a less punitive approach, since letting incarcerated people free may limit their risk of contracting the infection in prisons and can free up space inside these institutions to theoretically allow for some social distancing between remaining prisoners. Conversely, if releases (or even transfers between penal facilities) take place after the virus makes it into a prison, the penal institution could become a vector of transmission of the virus (Simpson and Butler, 2020). Notably, early release also would ideally involve welfare provision and support for those who leave prison in a context of heightened health risks, amid lockdown policies implemented to tackle the virus, and against the backdrop of the economic effects of such policies. Answers to our research questions will allow us to problematize how an acute global health challenge may reflect tensions produced by coexisting and sometimes contradictory policies designed to protect the general population, and the prison population as a part of the general population. More broadly, our analysis sheds light on the governance of vulnerable groups during a crisis.

## Methods and data

To explore what makes countries adopt policies designed to respond to the threat of COVID-19 in prisons, we compiled and analyzed data on 47 European countries between December 31st 2019 and June 1st 2020 from multiple sources, and applied event-history analysis. Our main sample consists of Council of Europe member states, and also includes Belarus and Kazakhstan in order to broaden the traditional penal comparative lens and allow for an analysis of similarities and differences among the successor states of the USSR. The majority of the countries we analyzed have national penal systems, while in some cases prisons are administered by region, including Germany (divided into prison administration by “Land” or state) and the United Kingdom (prison management is divided into England and Wales, Scotland, and Northern Ireland). For this reason, we use dates announced by the first region to implement each of the policies, in the event that the rest of the regions followed suit, or if national-level officials announced that such a policy would be recommended for other regions of the country.

### Outcomes

We analyzed two policies and their speed of their adoption: i) Date of first report on implementation of visitation rights limits at the national level; and ii) Date of first early releases or pardons at the national level. Information to detect both dates was gathered from government websites, as well as the websites of major prison associations, prison NGOs, and national and international media outlets (Appendix 1 contains all sources used for each country).

### Determinants of implementation of visitation rights limits and early releases or pardons

We use the following variables to explore what leads countries to adopt these two policies more rapidly, more slowly, or simply to reject them. While bans on visitation rights can be linked to more humane reasoning based on the idea that prisoners’ health must be protected from the threat of visitors bringing coronavirus into the prison, it can also be perceived as more punitive because visitation is an important right of prisoners. Conversely, it could be interpreted as normatively neutral, aimed at preventing the spread of the virus (even though the practical effects of this policy could lead to more humane or more punitive reverberations in the prison). Therefore our expectations regarding the variables allow for all of these interpretations; below we outline some of these possible expectations, though the review is not exhaustive. We also consider early release of prisoners to be a more humanitarian-oriented rather than punitive policy, which is reflected in our discussion.

#### Prison population rate

This variable measures the number of prisoners divided by the total population of each country. Countries with higher rates could be expected to limit visitation rights more rapidly, since this variable could be associated with nations more inclined to be “tougher on crime” and therefore less likely to recognize prisoners’ rights (Neil and Carmichael, 2015). On the other hand, prison population rates do not always correlate with quality of prison conditions, so countries could be slower to enact visitation bans if conditions of prisons allow for physically-distanced visits and for social distancing between prisoners within the prison. Releases and pardons may also be accelerated by higher prison population rates if these are combined with cramped prison conditions, yet higher incarceration rates may also be associated with more punitive systems which are unlikely to opt for early release of prisoners. These data have been obtained from the World Prison Brief (2020).

#### Percentage of foreign prisoners

This variable measures the proportion of foreign prisoners relative to the national prisoner population. Studies show that foreigners are overrepresented in prisons across much of Europe (Barker, 2018), and scholars have used the term “enemy penology” to refer to harsher sentences imposed on immigrants (McNevin, 2011). Vanessa Barker, for instance, has argued that the Nordic countries use prisons to exclude “outsiders” (foreign nationals, ethnic minorities, and racialized social groups) from the welfare state (Barker, 2017, 2018), thus challenging the idea that there is a trade-off between welfare spending and incarceration (Sutton, 2013; Wacquant, 2009). Countries with higher proportions of foreign prisoners may thus be faster to limit visitation rights, or less likely to concede early releases, since prisons in such systems may be used as a way to reinforce boundaries of membership and access to symbolic and material resources in the nation-state. Furthermore, criminal justice systems that have a larger proportion of foreigners in prisons may not implement pardons and early releases as readily for foreign prisoners due to legislation surrounding deportation of foreign nationals who have served a prison term. These data have been obtained from the World Prison Brief (2020).

#### Number of years since capital punishment was abolished

This variable measures the number of years since capital punishment was abolished. We use it to operationalize one component of punitiveness. Previous studies (Ruddell, 2005; Sutton, 2004) have suggested that countries with capital punishment are more likely to incarcerate at greater rates, signaling greater punitiveness. It is thus expected that countries in which capital punishment was recently abolished may be slower in limiting visitation rights, since adherence to this policy may represent a regime in which prisoners’ rights are more likely to be respected. Conversely, countries in which capital punishment has been recently abolished may accelerate visitation rights bans, since it could signal a propensity for the violation of prisoners’ rights. In terms of conceding pardons or early releases, the more time has passed since a country abolished capital punishment, the quicker it may be to release prisoners, since this measure can be taken as propensity for adherence to human rights. These data have been obtained from the Death Penalty Information Center (2020).

#### Prison occupancy level

This variable measures the capacity of each country to accommodate the total number of prisoners given its prison capacity. Research on the negative impact of overcrowding has been relatively consistent. Studies have shown, for instance, a direct and negative association between mental health outcomes of prisoners and overcrowding across countries (Fazel et al., 2017; Rabe, 2012). Studies on prisons conditions have also identified overcrowding to be a salient characteristic in understanding of how viruses spread among both prisoners and staff (Simpson et al., 2019), and between the prison and the broader community (Stuckler et al., 2008). As such, we expect that countries with higher occupancy rates could limit visitation rights more rapidly, since this measure may reduce contagion between the community and penal populations more promptly. In terms of conceding pardons or early releases, countries with higher prison occupancy levels may be quicker to free up prisons to avoid outbreaks in these facilities. These data have been obtained from the World Prison Brief.

#### Epidemic security index

This variable measures various institutional capacities countries have to tackle epidemics. We used national systematised information on emergency preparedness and response planning, exercising response plans, emergency response operation, linking public health and security authorities, risk communication, access to communications infrastructure, and trade and travel restrictions. Previous studies have suggested that the health system capacity of a country is associated with imprisonment (Schnittker et al., 2015; Testa, 2020). More specifically, researchers observe that countries in which health capacity is low are more likely to have higher incarceration rates. Thus, we expect this variable to increase the hazard rate of reporting bans on visitation rights, since these countries would have less capacity to attend to the prison population and to rapidly adopt measures associated with social distancing such as limiting visits. We also expect this variable to be associated with delaying early releases, since this could be a policy designed to avoid the increase of contagions. These data have been obtained from the Global Health Security Index (2020). To facilitate interpretation of this index we transformed values to z-scores.

### Control variables

We use the following control variables, since these could also be associated with speed of policy adoption.

#### Gross Domestic Product per capita

We employ a measure of gross domestic product (GDP) per capita (purchasing power parity for 2000 US$). Studies have detected that, with the exception of the United States and Japan, countries with higher levels of GDP per capita are less likely to implement harsh measures towards prisoners (Jacobs and Kleban, 2003; Miethe et al., 2005). However, other studies have not found this association to be consistent across countries (Ruddell, 2005; Weiss et al., 2020). Since previous results have suggested mixed findings, we do not expect a particular direction in the association. Furthermore, results associated with this variable could be of importance for scholars interested in measuring the overall association between GDP and prison policies when using meta-data analysis methods, as such researchers would be better positioned to ascertain whether results associated with GDP are conclusive. We log-transformed this variable to avoid influence of outliers because of the skewed distribution. These data have been obtained from the World Bank (2020).

#### Democracy index

We adopt a measure of democracy which identifies nations along a scale ranging from 0 (‘strongly autocratic’) to 100 (‘strongly democratic’). The Democracy Index is based on five categories: electoral process and pluralism; civil liberties; the functioning of government; political participation; and political culture. Studies on democracy and penality are inconclusive and show a plethora of possible relationships. Some studies have suggested that more democratic countries are more likely to be supportive of prisoners’ rights as part of a broader human rights framework (D’Amico and Williamson, 2015). As such, more democratic regimes may be more prone to accelerating visitation limits, because this could be taken as a way of protecting prisoners’ health during a pandemic. Alternatively, visitation rights may be limited more slowly in democratic states, as receiving visits may be deemed an important right for prisoners to exercise. By the same token, more democratic regimes could also implement early releases faster in order to uphold prisoners’ rights, or they can refrain from early release policy altogether if public opinion is on the more punitive side, or if policymakers stress punitiveness as a precondition for public safety before the electorate (Buntman, 2009). Data have been obtained from the Economic Intelligence Unit database for the year 2019 (Economist Intelligence Unit, 2020). To facilitate interpretation of this index we transformed values to z-scores.

#### Political orientation of dominant party

We classified the political orientation of the dominant party or coalition in each country’s government at the time of the pandemic, according to a right-to-left spectrum, by considering the last national election that determined the makeup of the current government for each country.^2^ We constructed a scale ranging from left to right using five values: 1 if a country had more than 45% of votes from a left-leaning party or coalition; 0.5 if a country had between 0 and 45% of votes from a left-leaning party or coalition; 0 if a center party or coalition won the election; –0.5 if a country had between 0 and 45% of votes from a right-wing party or a coalition; and –1 if a country had more than 45% of votes from a right-wing party or coalition. Sutton (2004) and Neil and Carmichael (2015) have suggested that governments’ political orientation has a bearing on incarceration. More specifically, higher rates of imprisonment are associated with the rule of right-leaning political parties rather than left-leaning ones. Sutton (2013), however, has found a weak association between left-leaning governments and penal trends. He shows that over time, the resistance of the left to incarceration weakened, with the rise of Clinton’s ‘third way’ strategy in the USA and Blair’s New Labour compromise in the UK. Overall, we could expect that left-leaning or left-center governments may be more prone to accelerate visitation rights limits because these measures may support prisoners’ rights during a pandemic; nevertheless, right-wing or right-center governments may also implement visitation bans faster because this measure could be understood as a harsh measure towards prisoners. By the same token, left-leaning or left-center governments could support early release policies since these measures could also signal support for prisoners’ rights. Data have been obtained from each election result published in each country.

### Methods

To obtain valid estimates to examine policy adoption, we employ event-history analysis, also known as survival analysis. This method allows us to explain events occurring in different countries over a specified period of time (Cleves, 2010). Event-history analysis has been used for various types of events ranging from decolonization (Strang, 1994) to policy adoption (Kogut and Macpherson, 2008). We particularly use the Weibull hazard function, since its ρ value can be used to interpret whether policy adoption significantly increases during the observed period. The Weibull function (h_0_(*t*)) is specified as h_0_(*t*)= ρ*t^ρ−1^. If ρ is less than 1, the speed of policy adoption (i.e. hazard of failure) decreases with time, while if it is greater than 1, the speed of the policy adoption increases with time. If an ongoing European or Council of Europe diffusion process is boosting the adoption of the two policies, a significant increase in the parameter of the models should be observed.

It is important to note that since outcomes could be a result of modeling countries as if they had been equally exposed, or not exposed at all, to the same risk at the same time, we defined two different onsets of risk: i) January 31st, 2020, when WHO declared COVID-19 to be a global health emergency; and ii) the first case detected in each country. Each onset of risk was used to predict each policy. Information to determine the first onset was derived from WHO’s press conferences (World Health Organization, 2020a, 2020b), and each first detected case per country was taken from the European Centre for Disease Prevention Control (European Centre for Disease Prevention and Control, 2020).

Since unobserved heterogeneity could also arise from information that countries share due to their regional closeness, implying that unobserved processes could bias the results of the parameters (Cleves, 2010) we adjusted the precision of the estimates for their adoption rates in reference to 6 sub-regional European clusters based on the United Nations geoscheme (World Population Prospects, 2015) (please see in Appendix 2 the regional cluster list with the countries). In other words, each sub-regional cluster was assigned a random effect—whose distribution does not depend on the observed variables—to model the potential impact of information exchange among countries within each cluster.

We carried out several sensitivity analyses to (1) indirectly assess whether the results were robust to model specification, and (2) using alternative distributions (exponential and Gompertz models) (Appendix 3). We also use logistic, Poisson and negative binomial models assuming that countries were independent of each other at the time of adopting policy of early releases or pardons (Appendix 4). We used Stata/SE 16.0 for all the analyses (StataCorp, 2015) (codes available in Appendix 5).

## Results

**Figure 1.**
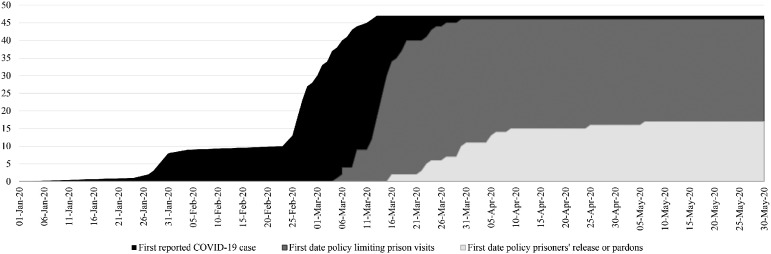
Accumulative of first COVID-19 cases, policies limiting prison visits and policies allowing early releases or pardons January 1st June 1st.

**Figure 2.**
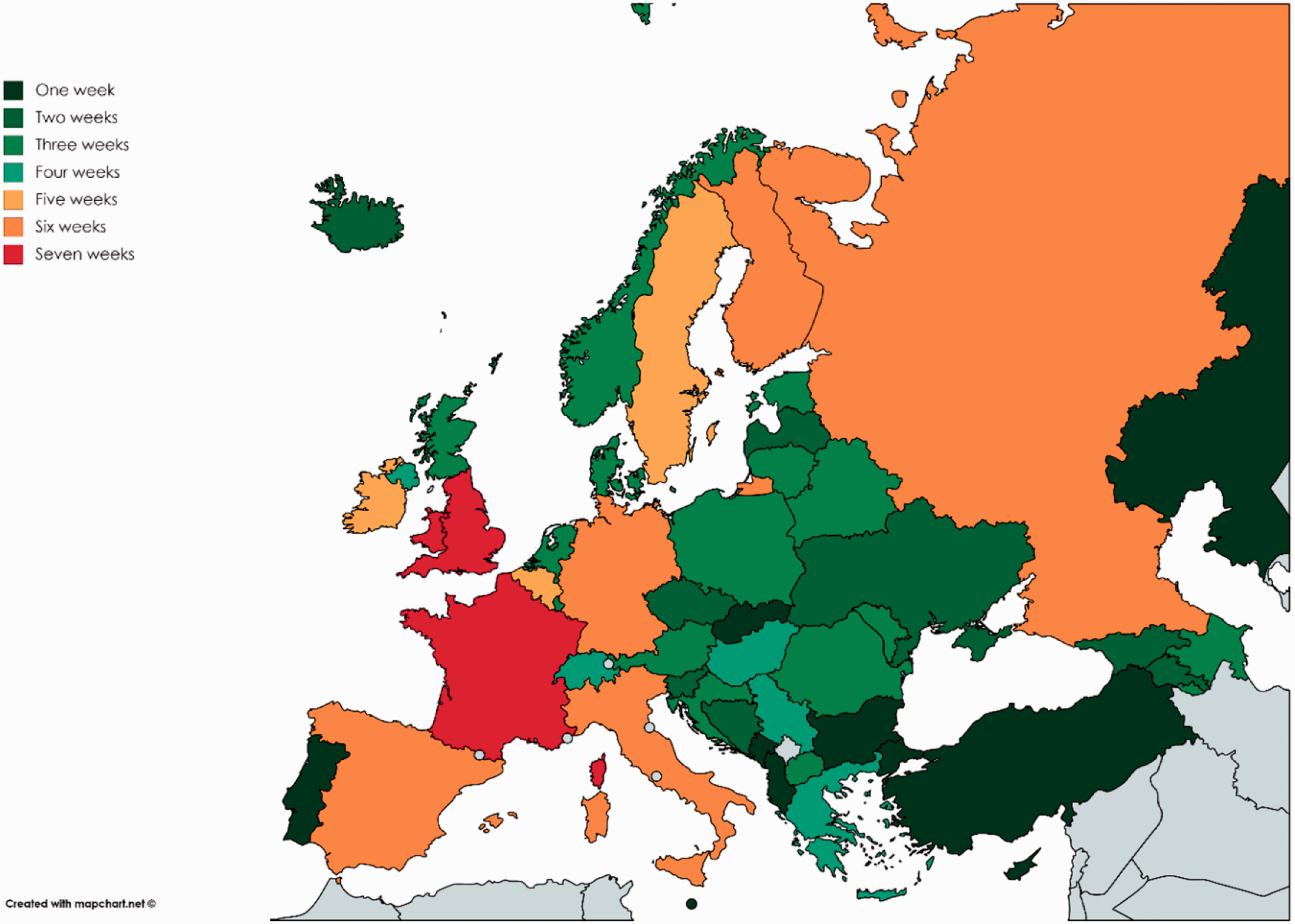
Number of weeks after each country limited visits to prisons after first case of COVID-19 was detected on each territory.

**Figure 3.**
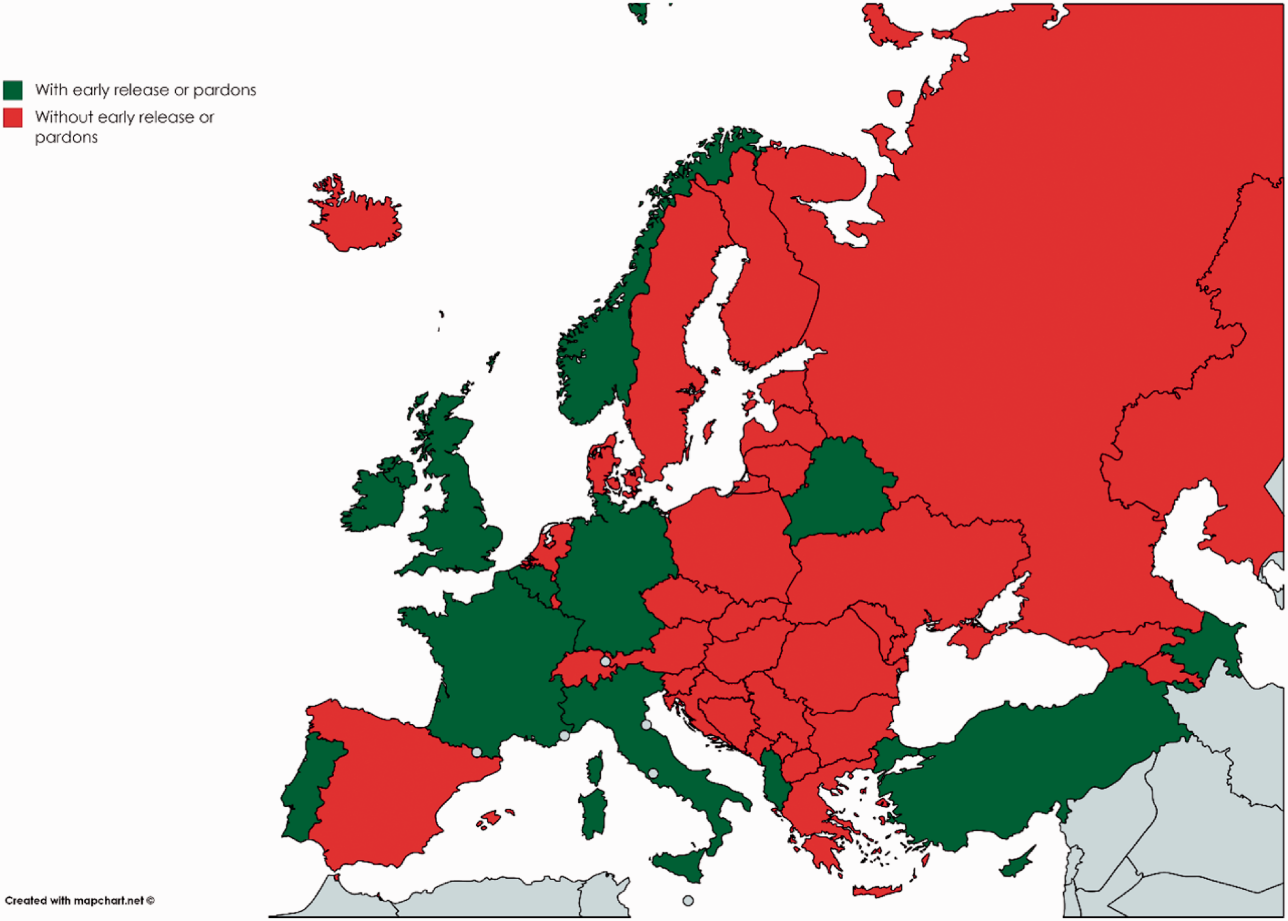
Countries which have implemented early release or pardons.

To tackle the COVID-19 pandemic in prisons, 47 Council of Europe member-states, as well as Belarus and Kazakhstan, implemented limitations on visitation rights, and only 16 of these implemented early releases or pardons. Figure 1 depicts the cumulative distribution of both policies from December 31st 2019 to June 1st 2020. In Table 1, we show the average of the number of days it took for a country to limit visitation rights, considering two dates: ‘January 31st 2020-WHO declares global health emergency,’ and ‘Respective date a country reports its first case of COVID-19.’ The average number of days in which countries implemented these policies were: 44 days (SD: 5·40) and 20 days (SD:13·90), respectively. Figure 2 depicts a map of Europe identifying for each country the number of weeks it took to limit visitation rights from the first reported case of COVID-19. It is noteworthy that Montenegro and Slovakia were the only countries that limited visitation before the first COVID-19 case was reported. Figure 3 shows the 16 countries that introduced early releases or pardons of prisoners. Using the same two dates, we observe in Table 1 the average number of days it took for countries to implement this policy: 58 days (SD:9) and 37 days (SD:19).

**Table 1.** Descriptive statistics of dependent and independent variables.

Variables	Mean	SD	Min	Max
***Outcomes***				
*Number of days since implementation of visitation rights limits was reported after January 31*st *2019 (WHO declares a Global International Emergency)*^a^	44.48	5.40	34	59
*Number of days since implementation of visitation rights limits was reported after first case was detected* ^a^	20.41	13.90	0	54
*Number of days since first early releases or pardons during pandemic at after January 31st * *2019(WHO declares a Global International Emergency)* ^a,^ ^b^	58.75	9.81	45	84
*Number of days since implementation of visitation rights limits was reported after first case was detected* ^a,^ ^b^	37.56	19.00	12	84
***Determinants***				
*Prison population rate*	139.06	77.21	37	97.4
*Percentage of foreign prisoners*	18.19	19.27	1.1	74.7
*Number of years since capital punishment was abolished*	26.45	15.52	0	92
*Prison occupancy level*	93.59	18.04	42.4	141.1
*Epidemic security index (z score)*	0.68	1.17	−1.15	3.47
***Control variables***				
*GDP per capita (ln)*	9.98	1.02	7.98	12.39
*Democracy index (z score)*	0.74	0.84	−1.34	1.95
*Political orientation of dominant party*	0.18	0.56	−1	1

^a^List with all sources is available in Appendix 1.

^b^This corresponds to the following 16 countries: Albania, Azerbaijan, Belarus, Belgium, Cyprus, France, Germany, Ireland, Italy, North Ireland, Norway, Portugal, Scotland, Slovenia, Turkey and United Kingdom.

### Spreading of the policy that limits visitation rights

Table 2 reports the structural parameter ρ in which the policy implementation of visitation rights limits is analyzed using two different onset risks. We observe that the structural parameter speed of adoption (ρ) capture increases of 16.90 (95% CI: 15·15, 18·84) for the first onset. This value indicates that the speed of detecting the first case of COVID-19 grows significantly over time. For instance, 45 days after the WHO declared the global emergency relative to 30 days after this declaration, countries were 630 times more likely to limit visitation rights (45/30)^16.90–1^). The structural parameter ρ for the second onset is 17·23 (95% CI: 13·62, 21·80) without the interaction effect between Percentage of foreign prisoners and Prison occupancy level, confirming that countries were rapidly and homogeneously responding to the pandemic. Lastly, the structural parameter ρ for this onset, in which an interaction effect has been introduced 15·95 (95% CI: 13·44, 18·94), also reinforces a convergence pattern across European countries.

**Table 2.** Survival models predicting ‘Date of first report on implementation of visitation rights limits at the national level.’

*Outcome*	Date of first report on implementation of visitation rights limits at the national level									
***Onset***	*January 31st, 2020-WHO declares global health emergency* ^a^			*Respective date a country reports its first case of COVID-19* ^b^						
***Determinants***	HR	95% CI		HR	95% CI		HR	95% CI		
*Prison population rate*	1.000	0.991	1.015	1.001	0.988	1.013	1.005	0.989	1.022
*Percentage of foreign prisoners*	1.004	0.986	1.022	1.003	0.984	1.023	**0.872**	**0.796**	**0.955**
*Number of years since capitalpunishment was abolished*	1.019	0.975	1.064	1.011	0.959	1.066	1.030	0.975	1.088
*Prison occupancy level*	0.987	0.966	1.008	0.989	0.966	1.013	**0.964**	**0.933**	**0.995**
*Epidemic security index (z score)*	**1.507**	**1.060**	**2.141**	**1.476**	**1.028**	**2.118**	1.380	0.965	1.088
*Percentage of foreign prisoners× Prison occupancy level*							**1.001**	**1.000**	**1.002**
***Control variables***									
*GDP per capita (ln)*	0.666	0.326	1.359	0.690	0.329	1.444	0.738	0.335	1.627
*Democracy (z score)*	1.561	0.567	4.294	1.372	0.480	3.925	1.523	0.461	5.023
*Political orientation of dominant party*	**3.199**	**1.842**	**5.556**	**3.095**	**1.741**	**5.502**	**3.049**	**1.767**	**5.250**
*Speed of adoption (ρ)*	**16.90**	**15.15**	**18.84**	**15.95**	**13.44**	**18.94**	**17.23**	**13.62**	**21.80**
*Number of countries*		43			41			41	
*Number of adoptions*		43			41			41	
*Time at risk*		1903			860			860	

^a^Countries in models Albania, Armenia, Austria, Azerbaijan, Belarus, Belgium, Bosnia and Herzegovina, Bulgaria, Croatia, Cyprus, Czech Republic, Denmark, Estonia, Finland, France, Georgia, Germany, Greece, Hungary, Iceland, Ireland, Italy, Kazakhstan, Latvia, Lithuania, Luxembourg, Malta, Montenegro, Netherlands, Norway Poland, Portugal, Romania, Russian Federation, Scotland, Serbia, Slovakia, Slovenia, Spain, Sweden, Switzerland, Turkey, Ukraine and United Kingdom.

^b^Same countries with the exception of Slovakia and Montenegro, since these countries limited visits before the first COVD-19 case was reported. HR: Hazard Ratio. Bold numbers indicate p < 0.05. All models control for country’s population.

In terms of variables that are associated with more or less rapid limits on visitation rights, we observe that one standard deviation decrease in the Epidemic security index accelerates the adoption of this policy by 49% (HR: 1·49 (95% CI: 1·06, 2·08)). The other determinants did not capture concomitant variation when analyzing how rapidly countries were limiting visitation rights. When observing the onset ‘Respective date a country reports its first case of COVID-19,’ results suggest that Prison population rate is not likely to be associated with speed variation of this policy. Prison occupancy level is associated with a 1.0% (HR: 0·99 (95%CI: 0·97, 1·00)) delay in visitation rights limits. Percentage of foreign prisoners is associated with an increase in the speed of policy implementation by 0.3% (HR: 1·00 (95% CI: 0·99, 1·02)). The variable Number of years since capital punishment was abolished is associated with an acceleration of this measure by 2.0% (HR: 1.02 (95% CI: 0.98, 1.06)). Alternatively, we also dichotomized this variable with presence or absence of capital punishment and results were similar (results are available in Appendix 6).

**Figure 4.**
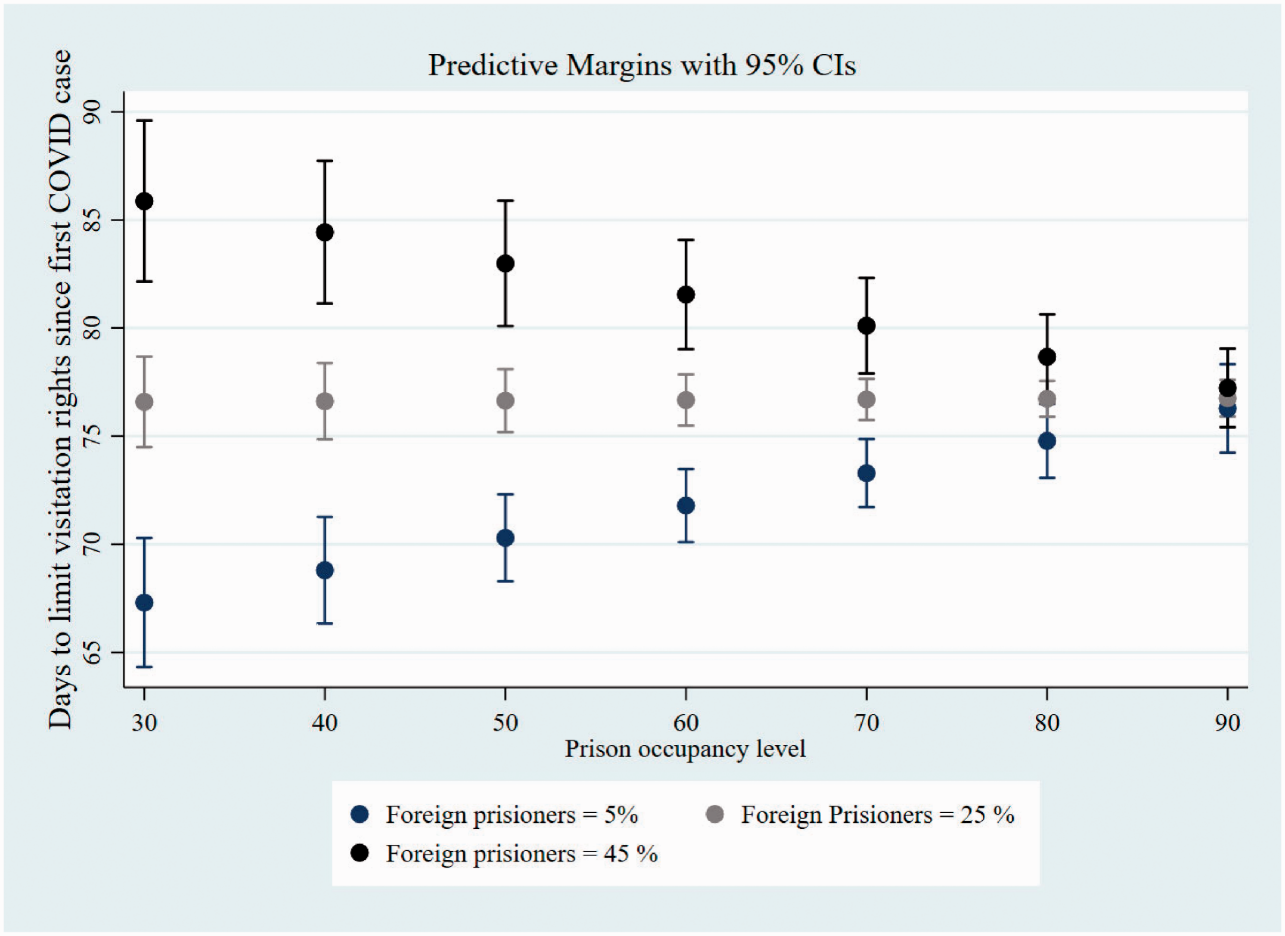
Predictive Number of days to limit visitations rights according to prison occupancy level and percentage of foreign prisoners.

However, once an interaction effect between Percentage of foreign prisoners and Prison occupancy level is introduced, we observe a difference in times to adopt this policy. In Figure 4 we plug the predictive margins of this interaction effect. We observe that, when Prison occupancy level is held constant at 30%, countries were faster to limit visitation rights by 18 days, if their foreign prison population was at 5% (68 days (95% CI:63.8, 71.8) relative to countries in which the foreign population was at 45% (85 days (95% CI:80.1, 90.18). The differences in adoption of this policy, considering differences between foreign prisoners, dilutes when the Prison occupancy level is higher than 70%.

Turning to the control variables, only Political orientation of dominant parties captures speed variation in this sample. Left-wing or left-center governments were accelerating the speed of visitation limits by 3 (HR: 3·09 (95% CI: 1·74, 5·50)) times.

### Adoption of early releases or pardons

In Table 3, we report results regarding early releases or pardons as policies adopted to curb the pandemic in the prisoner population. The structural parameter speed of adoption (ρ) captures a nonsignificant increase of 1·65 (95% CI: 0·87, 3·14) for the first onset, and 0.9 (95% CI: 0·22, 3·68) in the second onset analyzed. This indicates that across European countries and relative to visitation limits (as Figure 1 shows), early releases or pardons did not converge (as Figure 3 shows).

**Table 3.** Survival models predicting ‘Date of first early releases or pardons during pandemic at the national level.’

*Outcome*	Date of first early releases or pardons during pandemic at the national level					
***Onset***	*January 31st, 2020-WHO declares global health emergency* ^a^			*Respective date a country reports its first case of COVID-19*		
***Determinants***	HR	95% CI		HR	95% CI	
*Prison population rate*	0.997	0.991	1.002	0.999	0.994	1.005
*Percentage of foreign prisoners*	0.975	0.915	1.038	0.978	0.911	1.005
*Number of years since capital punishment was abolished*	0.979	0.879	1.091	0.988	0.890	1.097
*Prison occupancy level*	**1.066**	**1.027**	**1.107**	**1.070**	**1.031**	**1.110**
*Epidemic security index (z score)*	0.805	0.231	2.803	0.784	0.241	2.547
***Control variables***						
*GDP per capita (ln)*	4.514	0.261	77.879	3.834	0.157	93.054
*Democracy (z score)*	0.227	0.038	1.345	0.215	0.042	1.093
*Political orientation of dominant party*	0.492	0.049	4.025	0.543	0.071	4.153
*Speed of adoption (ρ)*	1.65	0.87	3.14	0.91	0.22	3.68
*Number of countries*		43			43	
*Number of adoptions*		14			14	
*Time at risk*	5336				4288	

^a^Countries in the models Albania, Armenia, Austria, Azerbaijan, Belarus, Belgium, Bosnia and Herzegovina, Bulgaria, Croatia, Cyprus, Czech Republic, Denmark, Estonia, Finland, France, Georgia, Germany, Greece, Hungary, Iceland, Ireland, Italy, Kazakhstan, Latvia, Lithuania, Luxembourg, Malta, Montenegro, Netherlands, Norway Poland, Portugal, Romania, Russian Federation, Scotland, Serbia, Slovakia, Slovenia, Spain, Sweden, Switzerland, Turkey, Ukraine and United Kingdom. HR: Hazard Ratio. Bold numbers indicate p < 0.05. All models control for country’s population.

Unlike visitation limits, the only characteristic associated with speed of adoption is Prison occupancy level. When we consider ‘Respective date a country reports its first case of COVID-19’ as the onset, this variable is associated with a 7% (HR: 1·07 (95% CI: 1·02, 1·11)) increase in accelerating the implementation of this policy. The Epidemic security index suggests that one standard deviation decrease in this variable reduces the speed of adoption of early releases or pardons, albeit nonsignificantly, by 19% (HR: 0·81 (95% CI: 0·23, 2·80). The other determinants, such as Percentage of foreign prisoners, Number of years since capital punishment was abolished, Prison population rate, and control variables GDP per capita, Democracy, and Political orientation of dominant parties are not likely associated with variation in the variable of time taken to adopt early releases or pardons. We also tested an interaction effect between Percentage of foreign prisoners and Prison occupancy level, and no significant associations were found (results available in Appendices 3, 4, and 6).

## Discussion

We present, to our knowledge, the most extensive study of COVID-19 policies in prison systems. We found that all Council of Europe member states, as well as Belarus and Kazakhstan, implemented prison lockdowns in the form of bans on visits. Another much-debated policy was early release and pardons of prisoners. Despite widespread discussion of decarceration to tackle the pandemic among scholars and civil society representatives around the world (Simpson and Butler, 2020; Strassle and Berkman, 2020), few countries opted for this policy. We observed that only 16 European countries took this path, and compared to the banning of visitation rights, implementation of this policy took longer. Our study points to variation in speed of policy adoption across Europe, and to understand this we explored different determinants. To sum up, we observed a case of full convergence when analyzing bans of visits, but policy divergence occurred in the case of early release and pardons. This raises important questions about whether the Council of Europe framework, or recommendations by international actors like the UNHCR or WHO, have sway over prison policy across Europe. The presence of these global bodies, which comparative penologists sometimes perceive as manifestations of the multi-level governance of prisons (Van Zyl Smit and Snacken, 2009; Vaughan and Kilcommins, 2007), have not resulted in similar responses to COVID-19 in the prisons of Europe, and local contexts of penality and punishment were more salient factors for policy-making. Speed variation in bans on visits and early releases or pardons may manifest contradictions and overlaps that exist between the idea of protecting prisoners from outbreaks, using the pandemic as a means to solve longstanding problems in the prison system, and ideas of prisoners as criminals from whom the rest of society must be defended, because they can become vectors of disease if released. In this sense, the pandemic offers new possibilities for yet another layer of othering of prisoners in public and political discourse. Moreover, release is a complicated process that may take longer and involve effective work of courts and the criminal justice system at large.^3^ It may also require the monitoring of quarantines, extra coronavirus testing, as well as welfare provision and support of former prisoners released into a pandemic context, with lockdowns and a struggling economy. When politicians consider these policies, they also keep in mind how the electorate or other constituents may respond. Thus, politics, welfare, public health, and humanitarian concerns all entwine here in non-straightforward ways.

Two findings of this study merit lengthier consideration. First, our study shows that when prison systems were overcrowded, countries were not more likely to delay or accelerate visit bans, but rather policy depended on who the prisoners were. This suggests a very imbricated public health strategy, since in uncertain times and across Europe certain types of penal population seem to be more protected than others. Indeed, when prisons are not overcrowded, but there are fewer incarcerated foreigners, countries are faster to limit visitation rights; but implementing this policy was much slower if the composition of the penal population had more than 45% foreigners and were not overcrowded.^4^ In short, while European countries homogeneously introduced banning visits as a policy to contain the virus, countries in which overcrowding interacted with foreign populations in prisons were not as quick to act.

If higher proportions of foreign prisoners in a country’s prisons may have delayed bans on visits under the circumstances of certain levels of overcrowding, two implications must be highlighted. First, this reinforces the notion that the relationship between punitiveness and health protection is not straightforward and is experienced unevenly across countries. Delaying bans on visits in countries in which the foreign prisoner population is high signals that eventual contagions between prison and general populations may not have been relevant to accelerating the implementation of this measure. Literature on penal populism, as well as penal nationalism, can be helpful here. If dominant state discourse stipulates that the general population must be protected from the threatening ‘other’ (Copson, 2014; Garland, 2001; Todd-Kvam, 2018), not prioritizing prisoners’ rights may be perceived as a rewarding policy to which public opinion may react favorably. An outcome of this approach in the context of a pandemic may be that the health of the general population suffers, since contagions are less likely to be prevented within a policy frame that does not prioritize the wellbeing of prisoners.

Second, our results suggest that countries were quicker to release or pardon prisoners if prisons were overcrowded. Differences could be explained by recognizing that in extreme circumstances, some countries are more likely to be guided by humanitarian and public health concerns in their policymaking, since COVID-19 would have direct consequences for large numbers of prisoners if it enters crowded prison spaces. However, in other cases, countries with overcrowded prisons may use the COVID-19 pandemic as an opportunity to reduce overcrowding, as a pandemic can produce a political window to do this quickly. Nevertheless, it is noteworthy that only 16 countries followed this path, perhaps suggesting that discourses about public safety that depict prisoners as dangerous criminals, which seem to be popular in many European countries (Brown and Pratt, 2000; Pratt, 2007; Simpson et al., 2019; Todd-Kvam, 2018), can easily overlap with narratives about public health. In this regard, European policymakers perhaps were publicly supporting the avoidance of early releases, since this measure could have mitigated risks associated with prisons becoming vectors of transmission of the virus into wider communities. Yet this measure also coincides with public safety concerns, in which prisoners are perceived as dangerous, and pardons would not be consistent with such a view of the “criminal” and the role of prisons in society. Lastly, in terms of what constitutes overcrowding it is important to bear in mind that more research is needed to assess whether current standards to determine prison capacity, for example the European Prison Rules or the Nelson Mandela Rules (United Nations, 2016), should be revisited in light of concerns about contagion of highly infectious disease that emerged over the course of the coronavirus pandemic.^5^ Other analyses should thus consider the extent to which thresholds of prison population density may not have been adequate to prevent COVID-19 outbreaks within these facilities.

Our discussion of these two findings must be interpreted with two caveats. First, it is important to recognize that prison overcrowding is a hotly contested issue (Simpson and Butler, 2020). There is no consensus on how prison overcrowding should be measured, and these numbers can be easily manipulated by authorities (Allen, 2010). In addition, the relationship between overcrowding and infectious and communicable diseases is still under-researched (Simpson et al., 2019). Crowding can also work very differently in different carceral settings: while crowding may result on one kind contagion dynamic in individual cellular accommodation, communal life in barracks typical of the Russian system and some postsocialist states (Badcock and Pallot, 2018; Pallot et al., 2009) would require very different strategies of enforcing social distancing, and infections may spread at a faster rate.

Second, since ethnic minority and foreign national populations in European countries have been disproportionately affected by prison preventive policies, then their ties with families over the course of prison lockdowns have been weakened, and therefore other negative outcomes, such as an increase in mental health illnesses in these populations, could also be expected (Shafran et al., 2020). However, the metric of foreign prisoners in a country’s prison is also a complex one and thus should be interpreted with care (see, for example, the discussion in Bhui, 2016). The metric can reflect a set of extremely differing naturalisation and citizenship policies in Europe, which may not be easily comparable across countries. While some scholars have used it as a proxy for ethnic minority prisoners, like Shammas (2015: 4) in the case of Norway, this brushes over the complex dynamics of racism, multiethnic identities, and variations in nationality policies. For example, while in some European countries ‘foreign nationals’ in prisons can include mostly first- or second-generation labor migrants, in other countries the numbers reflect a different dynamic. For instance, Estonia over-incarcerates its Russian-speaking population that holds so-called “grey passports” or alien passports: 35.5% of Estonian prisoners are classified as “foreigners” (World Prison Brief, 2020), most of whom are grey passport holders, compared with just over 6% of grey passport holders in the overall population (UNHCR, 2016). Roma, for example, are overincarcerated across Europe and face particular forms of discrimination, yet their citizenship status is a different matter from the extreme social exclusion they face. Roma can be citizens of the country they reside in, or can be included in the foreign national statistics in prisons if they are incarcerated in a country where they are not citizenship holders. Incarceration of foreigners is also shaped by prisoner transfer treaties between countries.

Our study has limitations that must be considered. First, in all country cases examined here, penal institutions can vary significantly from prison to prison and region to region. For example, an indicator such as “total occupancy level” for a country case may mask sizeable variation between different regions or different prisons within that country. Further, implementation of policy on the ground can also differ from policy proclamations. Thus the analysis only reflects general trends in a given penal policy at the national level, rather than its actual implementation. Second, a quantitative study of penal policy trends based on a select number of factors can divert attention away from the factors that politicians, policymakers, and other agents related to the prison system actually consider when making their decisions. For example, it is possible that prison services in countries with significant overcrowding in prisons pushed for early releases and pardons, making use of the opportunity presented by the pandemic to summon the political will to solve long-standing local problems. In addition, our analysis does not allow us to make explicit causal claims or to determine whether equifinality is at work (i.e. when differing processes lead to the same result). For example, one country can be driven by human rights considerations to release prisoners, while the government of another country may release prisoners due to significant overcrowding. Further qualitative analyses of policymaking mechanisms are thus needed to address these ambiguities.

Despite its limitations, the main contribution of the work is pointing in the direction of new questions about factors that shape how punishment is carried out, and how it works during a global crisis. We consider four possible directions to be particularly important. First, qualitative and quantitative work is needed for empirical investigation of how penal policies have potentially further marginalized incarcerated minority groups across Europe during the pandemic. Second, attention to additional levels of analysis is needed to capture regional variation within specific countries. While most of our sample is comprised of countries where decisions about lockdowns and releases were made at the national level, in the cases of Germany and Italy, for example, decisions were made by region, even if they followed national-level trends. It would be productive to disentangle patterns in countries where these two measures were decided at a subnational level, and then scaled to a national one (this discussion would contribute to questions on multi-level governance within nation-states, for example see Lodge and Wegrich, 2005). In addition to regional variation within a nation-state, another level of analysis could capture variation of policy implementation across penal institutions – a task riddled with methodological challenges. Third, while our preliminary analysis showed policy convergence with regard to visit bans, we should emphasize the need for greater precision and granularity when examining theses processes more critically. Future analyses should, for instance, attempt to better capture how different actors debated, delayed, and accepted this policy, with particular attention to how left/right dominant party orientation, broader societal characteristics, penal system characteristics, and the presence or absence of outbreaks within prisons informed these decisions (Okano and Blower, 2020).

Finally, several noteworthy trends emerged from our study that merit further inquiry from scholars of comparative penology interested in Nordic penal systems and the former socialist states of Central and Eastern Europe, especially from a penal nationalism perspective. With regard to the Nordic states, our study did not indicate policy convergence across these countries during the pandemic, despite the fact that they are often described in the literature as a cluster of states with exceptional penal features (Brangan, 2020; Ugelvik and Dullum, 2012). Our study thus challenges the ‘Nordic exceptionalism’ frame. In particular, the findings point to the need to analyse COVID-19 prison policies from the perspective of penal nationalism. Penal nationalism is defined by Vanessa Barker as “a form of state power that relies on the coercive tools and moral weight of criminal justice to respond to unwanted mobility in the service of national interests” (2018: 89). Barker draws on the case of Sweden and critiques the idea that the Nordic states are notoriously moderate in the use of penal power. She points to the over-incarceration of foreign nationals in Sweden (who make up 30 percent of the prison population compared with 8 percent of the general population), and argues that the Nordic welfare model, instead of being a universalist inclusionary project, in fact uses penal power to protect the welfare “bubble” from unwanted outsiders and to enforce welfare chauvinism (Barker, 2018: 90). Early release, then, may be unlikely in countries that exhibit this trend. Notably, our study showed that of the Nordic states, only Norway implemented early release in the timeframe we analyzed. Similar welfare-chauvinist trends can be inferred from research on Germany, where the narrative, material, and biopolitical exclusion of some migrants exists alongside the inclusion and reification of others as members of the German state who are given privileged access to welfare on account of their ‘Germanness’ (Zeveleva, 2017). In light of these theories, we can also probe the interaction effect we found between proportion of foreign prisoners and prison occupancy level, where countries were slower to limit visitation rights if they had a higher percentage of foreign prisoners in the event that occupancy level was below 70%. Perhaps we can interpret this finding as a manifestation of penal nationalism, if protection of prisoners from the threat of infection coming in from the outside was not prioritized at a national level, especially if this is combined with a reluctance to release prisoners.

However, if we turn to the work of Lynne Haney (2016) on Central European countries and employ a broader definition of penal nationalism, we can say this phenomenon sees politicians and policymakers harnessing penal power with the aim of securing three things: 1) national welfare for ‘insiders’ (as argued above); 2) the sovereignty of national governments from external pressures, such as those emanating from international human rights groups or EU bodies (in the case of Central European countries); 3) the discursive link drawn by political elites between the general problem of crime with historical crimes of particular ethnic or national groups against the nation (Haney 2016: 357). This analytical lens may allow us to begin to make sense of the cluster of country cases that emerges east of Germany, where visits were banned quicker than in the rest of Europe, and where early releases and pardons were less likely to take place than in the rest of Europe. In her work, Haney analyses Poland, Hungary, the Czech Republic, and Slovakia to understand why these states have higher imprisonment rates than countries to the West, outpaced only by some countries further East like Russia and Ukraine (Haney 2016: 349). She points to the coexistence of high imprisonment rates in these countries with some of the lowest official crime rates in Europe, alongside high fears of crime (Haney 2016: 351). Haney argues that in addition to safeguarding welfare from ‘othered’ minorities (most prominently, the racialized Roma), penal nationalism has been deployed in official political rhetoric and penal policy in Central Europe to turn crime control into a topic central for national sovereignty. This has manifested itself in resistance to pressures from the Council of Europe, the European Court of Human Rights, and the Committee for the Prevention of Torture over several of the penal policies in these countries. As is often the case in comparative penology and in the sociology of punishment, countries yet further East have received even less attention in comparative perspective, and there is much work to be done on examining whether and why penal policies in these countries diverge, converge, or harmonize with European recommendations. Following Haney’s warnings, in this case we must be wary of attributing certain cultures of harsh punishment to a historical legacy of socialism, and incremental analysis of policy and practice should guide our inquiry.

## Conclusion

In light of the global nature of the COVID-19 pandemic, international organizations such as the WHO recommended a comprehensive approach to preventing outbreaks in prisons, including early releases and banning of visits in a way that took prisoners’ rights into account. Our findings emphasise that all European countries banned visits, yet only 16 introduced early releases or pardons. The analysis points to two major findings:

1. Countries with prison overcrowding problems were quicker to release or pardon prisoners;
2. When prisons were not overcrowded, countries with higher proportions of local nationals in their prisons were much faster to limit visits relative to prisons in which the foreign population was high.

Our work highlights the need for continued research to understand how different national responses may have affected the overall health and wellbeing of prisoners, and whether and how policies aimed at tackling the pandemic further marginalize already vulnerable people. If we assume that releases indicate a welfare approach (especially in countries where prisoners who qualify for early release during the pandemic also receive access to social services), while lack of release indicates a propensity for continued incarcerate despite the pandemic threat within prisons, then this dichotomy can echo the idea discussed by Wacquant (2009) and Sutton (2013) that there is a trade-off between welfare and incarceration in society. However, if we employ the analytical lens of penal nationalism, we can see that the welfare-incarceration dichotomy is problematized if we take into account the fact that incarceration in many countries disproportionately affects foreign nationals and ethnic minorities, as Vanessa Barker has argued in the case of Sweden (2017, 2018). In this way, it may be productive to view a slow implementation of banning prison visits as a manifestation of penal nationalism, in the event that this showed that protecting prisoners from infections coming in from outside was not a priority, combined with a reluctance to release prisoners.

Our study contributes to discussions of penal nationalism in two different ways: first, our findings that Nordic states, with the exception of Norway, did not opt for early releases, and that higher proportions of foreigners in prisons delayed visitation bans, may be part of the universe of ideas and policies that protect national welfare for ‘insiders’ while deploying penal power to cut off ‘outsiders’ at the price of not controlling conditions of spreading viruses. Second, the lack of influence of international bodies that partake in multi-level governance of prisons (especially those at the EU level) may be attributed to the type of penal nationalism that mobilizes discourses and prison policies in order to emphasize national sovereignty and resist international pressures, as Haley (2016) found to be typical for the Central European states of Poland, Hungary, the Czech Republic, and Slovakia.

## Supplementary Material

Supplementary material

## Notes

1 Preliminary analysis that lay the foundation for this study was conducted by Olga Zeveleva in spring 2020, when monitoring prison policy responses to the COVID-19 pandemic across 76 countries around the world as part of the ERC-funded research project GULAGECHOES (grant agreement ID 788448). The resulting original database of prison policies showed that the most common and comparable responses, which were also reported on by prison services, NGOs, and the media around the world (and thus were available for analysis), included: 1) limitations on visitation rights (by March 31, 2020, 66 countries had implemented this); 2) the second most common response was release of prisoners (by 31 March, 2020, 23 countries had done this or were about to do so). These findings are partly summarized in the GULAGECHOES research blog post available at https://blogs.helsinki.fi/gulagechoes/2020/04/01/coronavirus-in-prisons-a-global-perspective-tracking-policy-responses-releases-and-riots/. This preliminary work informed the selection of policies analyzed in this article. Major sources of information included NGO reports, prison services reports, and the media in English, Russian, German, French, and other languages using website translation services. We are particularly grateful to Prison Insider (https://www.prison-insider.com/en/articles/coronavirus-la-fievre-des-prisons#europe-5e7254d1cebfe), and the World Prison Brief (https://www.prisonstudies.org/news/international-news-and-guidance-covid-19-and-prisons-13-march-30-november). The present study brought the database of policy responses up to date and zoomed in on Europe.

2 It is important to note that our operationalization of this variable reflects only part of the political climate in a given country. To identify political ideology variation more comprehensively, it would be helpful to consider additional dimensions relating to populism, nationalism, pro-status-quo sentiments, and the liberal-illiberal divide in future works.

3 Pardons, in contrast to early releases, are usually either part of a well-established tradition or heavy use of executive power. In our analysis, we do not distinguish between early releases and pardons, though this could be a task for further qualitative inquiry.

4 Perhaps this could also be connected to the fact that it may be more likely for greater numbers of foreign prisoners to have family abroad.

5 The Nelson Mandela Rules, adopted by the UN in 2015, set the standard minimum rules for the treatment of prisoners and give guidance on various aspects of prison management, including some guidance on space that should be allocated per prisoner. They are not legally binding.

6 This corresponds to the actual exponent to the fraction 45/30, to obtain the speed across the groups when these are compared.
