# Supplementary material for: COVID-19 and European carcerality: Do national prison policies converge when faced with a pandemic?
Source: Punishm Soc. 2022 Oct;24(4):642–66. doi: 10.1177/14624745211002011 (PMC9464929; doi:10.1177/14624745211002011)
Supplement: Supplementary material [file Appendices.pdf]

Supplementary material

Appendix 1

Table A1.1 Sources used for identifying 'Date of first report on implementation of visitation rights limits at the national level' and 'Date of first early release or pardon during pandemic at the national level', records for European countries between December 31st 2019 and June 1st 2020.

**Country**	**Date of first report on implementation of visitation rights limits at the national level**	Link 1 (enough if the information is from an official source or the NGO Prison Insider)	Link 2 (supplementary link, if available)	**Date of first early release or pardon during pandemic at the national level**	Link 1	Link 2 (if available)
Albania	14/03/2020	https://www.europris.org/general-directorate-of-prisons-al/	https://www.europris.org/wp-content/uploads/2020/04/Albanian-Prison-System-COVID19.pdf	24/03/2020	https://www.prison-insider.com/en/articles/europe-coronavirus-la-fievre-des-prisons	https://balkaninsight.com/2020/03/24/albania-to-free-600-prisoners-as-pandemic-precaution/
Armenia	25/3/2020	https://news.am/rus/news/568201.html	https://newsarmenia.am/news/armenia/v-armyanskikh-tyurmakh-provedeny-testy-na-koronavirus-/	**No**	http://www.justice.am/ru/article/2699	https://www.kavkaz-uzel.eu/articles/348724/
Austria	15/03/2020	https://www.europris.org/wp-content/uploads/2020/04/Ministry-of-Justice-AT.pdf	https://www.prison-insider.com/en/articles/europe-coronavirus-la-fievre-des-prisons#autriche-5e84d42b3b630	**No**		
Azerbaijan	17/03/2020	https://cpj.org/blog/2020/03/journalist-afgan-mukhtarli-azerbaijani-prisoners-a.php	https://media.az/society/1067775962/u-33-zaklyuchennyh-v-azerbaydzhane-vyyavlen-koronavirus/	6/4/2020	https://apa.az/en/domestic-news/Azerbaijani-president-signed-a-Decree-pardoning-176-prisoners-aged-over-65	https://azertag.az/en/xeber/President_Ilham_Aliyev_issues_Decree_to_pardon_group_of_convicts
Belarus	16/3/2020	https://spring96.org/ru/news/96246	https://www.belta.by/society/view/v-mestah-lishenija-svobody-vremenno-otmenili-svidanija-s-posetiteljami-383446-2020/	4/5/2020 (annual amnesty)	https://rg.ru/2020/05/04/bolee-25-tysiach-osuzhdennyh-belarusi-vyshli-na-svobodu-po-amnistii.html	
Belgium	14/3/2020	https://www.europris.org/wp-content/uploads/2020/03/ENG-Information-visitors-13-03.pdf		31/3/2020	https://www.brusselstimes.com/belgium/103626/coronavirus-	

					prompts-calls-to-free-inmates-to-ease-overcrowding-in-belgian-prisons-covid19/	
Bosnia and Herzegovina	13/3/2020	https://detektor.ba/2020/03/24/u-zatvorima-u-bih-zabranjene-posjete-zbog-pandemije-i-uvedene-preventivne-mjere/	https://faktor.ba/vijest/zbog-koronavirusa-zabranjene-posjete-i-dopusti-iz-zatvora-cijeli-objekat-dezinficiran/74025	**No**		
Bulgaria	15/3/2020	https://www.europris.org/general-directorate-execution-of-sentences-bg/	http://www.focus-news.net/news/2020/03/15/2757297/ministar-danail-kirilov-v-zatvorite-sa-zabraneni-vsichki-poseshteniya-i-izvezhdaneto-na-lishenite-ot-svoboda-izvan-sgradite.html	**No**		
Croatia	14/3/2020	https://www.europris.org/wp-content/uploads/2020/04/Covid-19-measures-and-activities-Croatia.pdf		**No**		
Cyprus	15/3/2020	https://cyprus-mail.com/2020/04/17/coronavirus-nicosia-prisons-lays-out-measures-taken-since-march-10/		27/3/2020	https://cyprus-mail.com/2020/03/27/coronavirus-around-50-prisoners-to-be-released-reports/	
Czech Republic	14/3/2020	https://www.europris.org/prison-service-of-the-czech-republic-cz__trashed/		1/0/1900	https://www.lidovky.cz/domov/tuzemskym-veznicim-se-koronavirus-zatim-vyhyba-trestanci-opatreni-respektuji-nikde-neprobihaji-vetsi.A200502_105522_ln_domov_rkj	
Denmark	18/3/2020	https://www.kriminalforsorgen.dk/corona/		No		

Estonia	14/03/2020	https://www.europris.org/estonian-prison-service-ee-covid19/		**No**		
Finland	13/03/2020	https://www.rikosseuraamus.fi/fi/index/ajankohtaista/tiedotteetjauutiset/2020/03/rikosseuraamuslaitosottaakayttoonsuomi.fi-viestit-sahkoinenviranomaispostisaavuttaanytmyosvangit.html		No		
France	18/03/2020	https://www.europris.org/ministry-of-justice-department-of-prisons-fr/	https://www.prison-insider.com/en/articles/europe-coronavirus-la-fievre-des-prisons	23/03/2020	https://www.francetvinfo.fr/sante/maladie/coronavirus/coronavirus-le-ministere-de-la-justice-va-autoriser-la-liberation-de-5000detenus-en-fin-de-peine_3881053.html	https://www.france24.com/en/20200406-europe-worries-about-virus-timebomb-in-its-prisons
Georgia	5/3/2020	https://www.europris.org/wp-content/uploads/2020/03/Info_for_EUROPRIS_Georgia-_MOJ_SPS_Covid-19.pdf		No		
Germany	9/3/2020	https://www.prison-insider.com/en/articles/europe-coronavirus-la-fievre-des-prisons	https://www.vice.com/de/article/qjdxx3/coronavirus-covid-19-gefangnisse-sars-cov-2	22/03/2020	https://www.prison-insider.com/en/articles/europe-coronavirus-la-fievre-des-prisons	
Greece	19/3/2020	https://www.prison-insider.com/en/articles/europe-coronavirus-la-fievre-des-prisons#grece		No		
Hungary	27/3/2020	https://www.helsinki.hu/en/the-situation-in-hungarian-prisons-in-light-of-covid-19/	https://www.helsinki.hu/en/the-situation-in-hungarian-prisons-in-light-of-covid-19-2/	No	https://www.prison-insider.com/en/articles/europe-coronavirus-la-fievre-des-prisons#hongrie	
Iceland	6/3/2020	https://www.mbl.is/frettir/innlent/2020/04/15/aldrei_minna_um_fikniefni/	https://www.mbl.is/mogginn/bladid/innskraning/?redirect=%2Fmogginn%2Fbladid%2Fgre	No		

			in%2F1750147%2F%3Ft%3D894297102&page_name=grein&grein_id=1750147			
Ireland	27/03/2020	https://www.independent.ie/world-news/coronavirus/prisoners-offered-virtual-visits-from-loved-ones-after-all-in-person-visits-suspended-39081343.html	https://www.iprt.ie/latest-news/irish-prison-service-and-covid-19/	30/03/2020	https://www.justice-ni.gov.uk/news/covid-19-temporary-release-prisoners-scheme	
Italy	8/3/2020	http://www.prisonobservatory.org/upload/25032020European_prisons_during_covid19.pdf	https://www.aljazeera.com/news/2020/03/inmates-die-prison-riots-coronavirus-rules-italy-200309125813658.html	16/3/2020	https://www.hrw.org/news/2020/03/20/wider-steps-needed-protect-prisoners-health-italy	https://www.france24.com/en/20200318-italy-must-release-inmates-to-fight-virus-in-prison-rights-group
Kazakhstan	16/3/2020	https://tengrinews.kz/kazakhstan_news/v-koloniyah-zapretili-svidaniya-s-osujdennyimi-394828/	https://polisia.kz/ru/kakie-mery-profilaktiki-covid-19-prinimayut-v-tyur-mah-pavlodarskoj-oblasti/	**No**		
Latvia	12/3/2020	https://www.europris.org/wp-content/uploads/2020/03/Latvian-Prison-Administration.pdf	https://www.prison-insider.com/en/articles/europe-coronavirus-la-fievre-des-prisons#lituanie-5e90a73ceecb3	**No**		
Lithuania	19/03/2020	https://www.europris.org/ministry-of-justice-prison-department-lt/		**No**		
Luxembourg	18/03/2020	http://www.prisonobservatory.org/index.php?option=com_content&view=article&id=32:covid-19-what-is-happening-in-european-prisons&catid=7&Itemid=101	https://ap.gouvernement.lu/fr/actualites.gouvernement%2Bfr%2Bactualites%2Btoutes_actualites%2Bcommuniques%2B2020%2B03-mars%2B03-admpenitentiaire-renforcement-.html	No	https://www.bloomberg.com/news/articles/2020-03-26/prisoners-riot-in-luxembourg-amid-restrictions-on-visitors	
Malta	12/3/2020	https://www.maltatoday.com.mt/news/national/100975/coronavirus_no_visitors_in_prison_and_detention_centres_for_next_seven_days#.Xr2EBWgzZPY		**No**		
Montenegro	16/3/2020	http://www.rtcg.me/vijesti/drustvo/272249/zatvaraju-se-skole-i-vrtici-zabranjena-okupljanja.html		No (debates ongoing at the time of research)	http://www.rtcg.me/vijesti/drustvo/278010/vlada-podrzala-	

					predlozeni-zakon-o-amnestiji.html	
Netherlands	14/3/2020	http://www.prisonobservatory.org/upload/03042020European_prisons_during_covid19.pdf		No		
Norway	12/3/2020	https://www.kriminalomsorgen.no/tiltak-som-gjelder-fengsel.525465.no.html		16/3/2020	http://www.prisonobservatory.org/upload/17042020European_prisons_during_covid19%233.pdf	
Poland	19/03/2020	http://www.prisonobservatory.org/upload/17042020European_prisons_during_covid19%233.pdf#page=12		No		
Portugal	8/3/2020	http://www.prisonobservatory.org/upload/25032020European_prisons_during_covid19.pdf		9/4/2020	https://uk.reuters.com/article/us-health-coronavirus-portugal/portugal-suspends-water-electricity-shutoffs-and-releases-some-prisoners-amid-coronavirus-outbreak-idUKKCN21Q38F	https://www.portugalresident.com/judges-call-on-government-to-release-1400-prisoners/
Republic of Moldova	13/03/2020	https://www.europris.org/wp-content/uploads/2020/03/COVID-19-Moldova.pdf		No	https://promolex.md/17734-apel-cu-privire-la-implementarea-masurilor-urgente-si-suplimentare-pentru-sistemul-penitenciar/?lang=ro	
Romania	15/3/2020	https://www.europris.org/wp-content/uploads/2020/04/Addressing-Corona-virus-in-European-prisons-NAP.pdf		No		
Russian Federation	16/03/2020	http://fsin.gov.ru/news/index.php?ELEMENT_ID=502612	https://ria.ru/20200318/1568789372.html	No		

Serbia	30/03/2020	https://javno.rs/analiza/karantinski-zivot-u-zatvoru	https://www.021.rs/story/Info/Srbija/238374/Zabranjene-posete-zatvorenicima-u-naredne-dve-nedelje.html	No		
Slovakia	6/3/2020	https://www.europris.org/general-directorate-of-the-corps-of-prison-and-court-guard-sk/		No		
Slovenia	13/3/2020	https://www.gov.si/novice/2020-03-13-obvestilo-obiskovalcem-zaprtih-oseb-v-zavodih-za-prestajanje-kazni-zapora-in-prevzgojnem-domu-dopolnilo/		30/3/2020	https://siol.net/novice/slovenija/iz-zapora-zacasno-izpustili-68-zapornikov-522062	
Spain	15/3/2020	https://elpais.com/sociedad/2020-03-09/interior-impone-restricciones-en-las-carceles-tras-detectar-un-caso-de-coronavirus-y-cuatro-sospechosos.html	https://elpais.com/espana/2020-03-15/prisiones-suspende-las-visitas-y-los-permisos-a-los-reclusos-por-el-coronavirus.html	No		
Sweden	12/3/2020	https://www.kriminalvarden.se/om-kriminalvarden/nyheter/2020/mars/stopp-for-besok-permission-och-egen-installelse/		No		
Switzernland	25/3/2020	https://www.cdt.ch/ticino/nelle-prigioni-ticinesi-ora-piu-soli-che-mai-EC2499042	http://www.prisonobservatory.org/upload/03042020European_prisons_during_covid19.pdf	No		
North Macedonia	12/3/2020	https://tv21.tv/mk/zatvorite-vo-makedonija-pod-posebni-merki-zabraneti-se-poseti-i-napushtane-na-zatvorot-za-vikend-poradi-koronavirusot/		No		
Turkey	16/3/2020	https://www.al-monitor.com/pulse/originals/2020/03/turkey-coronavirus-spread-case-triple.html		23/3/2020	https://www.middleeasteye.net/news/coronavirus-turkey-law-release-inmates-prisons	
Ukraine	13/3/2020	https://minjust.gov.ua/news/ministry/evgeniy-gorovets-zaprovadjeno-obmejennya-na-vidviduvannya-ustanov-vikonannya-pokaran-ta-sizo		6/5/2020	https://lb.ua/pravo/2020/05/07/457038_komitet_radi_podderzhal_amnistiyu.html	https://iportal.rada.gov.ua/news/Novyny/192445.html

United Kingdom – England and Wales	24/3/2020	https://www.gov.uk/government/news/prison-visits-cancelled		24/4/2020	https://www.gov.uk/government/publications/covid-19-prison-releases	
United Kingdom – Northern Ireland	23/3/2020	https://www.derryjournal.com/news/uk-news/all-prison-visits-suspended-northern-ireland-minimise-contagion-risk-2504421		30/3/2020	https://insidetime.org/northern-ireland-justice-minister-showing-rest-of-the-uk-the-way-on-prisons-virus-policy/	
United Kingdom - Scotland	24/3/2020	https://www.prison-insider.com/en/articles/europe-coronavirus-la-fievre-des-prisons#ecosse		4/5/2020	https://www.gov.scot/news/short-term-prisoner-release/	

Appendix 2

Appendix 2 List with United Nations regional clusters and countries

This list is based on United Nations' classification (United Nations 2015).

Europe

Eastern Europe

Belarus, Bulgaria, Czech Republic, Hungary, Poland, Republic of Moldova, Romania, Russian Federation, Slovakia, and Ukraine.

Northern Europe

Denmark, Estonia, Finland, Iceland, Latvia, Lithuania, Norway, Sweden, Northern Ireland, Ireland, England, and Scotland.

Southern Europe

Albania, Andorra, Bosnia and Herzegovina, Croatia, Greece, Italy, Kosovo, Malta, Montenegro, Portugal, Serbia, Slovenia, Spain, and North Macedonia.

Western Europe

Austria, Belgium, France, Germany, Luxembourg, Netherlands, and Switzerland.

Central Asia

Kazakhstan.

Western Asia

Armenia, Cyprus, and Georgia.

Appendix 3

Table A3.1 Survival models predicting ‘Date of first report on implementation of visitation rights limits at the national level.’ (Gompertz models).

Outcome	**Date of first report on implementation of visitation rights limits at the national level**								
Onset	*January 31st, 2020-WHO declares global health emergency* [a]			*Respective date a country reports its first case of COVID-19* [b]					
Determinants	HR	95% CI		HR	95% CI		HR	95% CI	
Prison population rate	1.003	0.991	1.015	1.001	0.989	1.013	1.006	0.989	1.022
Percentage of foreign prisoners	1.005	0.987	1.023	1.004	0.985	1.024	**0.872**	**0.796**	**0.955**
Number of years since capital punishment was abolished	1.019	0.976	1.063	1.011	0.960	1.064	1.030	0.975	1.087
Prison occupancy level	0.987	0.967	1.007	0.990	0.967	1.012	**0.964**	**0.933**	**0.996**
Epidemic security index (z score)	**1.507**	**1.061**	**2.142**	**1.476**	**1.029**	**2.118**	1.391	0.966	2.004
Percentage of foreign prisoners x Prison occupancy level							**1.001**	**1.000**	**1.003**
Control variables									
GDP per capita (ln)	0.683	0.335	1.391	0.703	0.339	1.459	0.752	0.343	1.650
Democracy (z score)	1.527	0.552	4.228	1.346	0.474	3.820	1.504	0.455	4.970
Political orientation of dominant party	**3.200**	**1.843**	**5.556**	**3.074**	**1.717**	**5.501**	**3.049**	**1.768**	**5.260**
Population size (ln)	1.202	0.928	1.556	1.164	0.824	1.643	1.162	0.859	1.573
Number of countries		43			41			41	
Number of adoptions		43			41			41	
Time at risk		1903			860			860	

[a] Countries in models Albania, Armenia, Austria, Azerbaijan, Belarus, Belgium, Bosnia and Herzegovina, Bulgaria, Croatia, Cyprus, Czech Republic, Denmark, Estonia, Finland, France, Georgia, Germany, Greece, Hungary, Iceland, Ireland, Italy, Kazakhstan, Latvia, Lithuania, Luxembourg, Malta, Montenegro, Netherlands, Norway Poland, Portugal, Romania, Russian Federation, Scotland, Serbia, Slovakia, Slovenia, Spain, Sweden, Switzerland, Turkey, Ukraine and United Kingdom. [b] Same countries with the exception of Slovakia and Montenegro, since these countries limited visits before the first COVD-19 case was reported. HR: Hazard Ratio. Bold numbers indicate $p < 0.05$.

Table A3.2 Survival models predicting 'Date of first report on implementation of visitation rights limits at the national level.' (Exponential models)

Outcome	**Date of first report on implementation of visitation rights limits at the national level**								
Onset	*January 31st, 2020-WHO declares global health emergency* [a]			*Respective date a country reports its first case of COVID-19* [b]					
Determinants	HR	95% CI		HR	95% CI		HR	95% CI	
Prison population rate	1.000	0.999	1.001	1.000	0.995	1.004	1.000	0.995	1.004
Percentage of foreign prisoners	0.999	0.997	1.002	1.000	0.992	1.009	1.013	0.984	1.043
Number of years since capital punishment was abolished	1.001	0.999	1.004	0.997	0.986	1.008	0.996	0.985	1.007
Prison occupancy level	0.999	0.998	1.001	1.002	0.988	1.016	1.004	0.991	1.018
Epidemic security index (z score)	**1.028**	**1.004**	**1.053**	1.122	0.927	1.358	1.129	0.941	1.354
Percentage of foreign prisoners x Prison occupancy level							1.000	1.000	1.000
Control variables									
GDP per capita (ln)	0.965	0.883	1.055	0.710	0.463	1.090	0.706	0.462	1.078
Democracy (z score)	1.046	0.949	1.153	1.073	0.552	2.084	1.066	0.549	2.071
Political orientation of dominant party	**1.090**	**1.050**	**1.132**	0.962	0.626	1.479	0.967	0.638	1.467
Population size (ln)	1.004	0.989	1.019	**0.784**	**0.685**	**0.896**	0.785	0.688	0.896
Number of countries		43			41			41	
Number of adoptions		43			41			41	
Time at risk		1903			860			860	

[a] Countries in models Albania, Armenia, Austria, Azerbaijan, Belarus, Belgium, Bosnia and Herzegovina, Bulgaria, Croatia, Cyprus, Czech Republic, Denmark, Estonia, Finland, France, Georgia, Germany, Greece, Hungary, Iceland, Ireland, Italy, Kazakhstan, Latvia, Lithuania, Luxembourg, Malta, Montenegro, Netherlands, Norway Poland, Portugal, Romania, Russian Federation, Scotland, Serbia, Slovakia, Slovenia, Spain, Sweden, Switzerland, Turkey, Ukraine and United Kingdom. [b] Same countries with the exception of Slovakia and Montenegro, since these countries limited visits before the first COVD-19 case was reported. HR: Hazard Ratio. Bold numbers indicate $p < 0.05$.

Appendix 4

Table A4.1 Survival models predicting 'Date of first early releases or pardons during pandemic at the national level.'(Gompertz models)

Outcome	**Date of first early releases or pardons during pandemic at the national level**								
Onset	*January 31st, 2020-WHO declares global health emergency* [a]			*Respective date a country reports its first case of COVID-19* [b]					
Determinants	HR	95% CI		HR	95% CI		HR	95% CI	
Prison population rate	0.997	0.003	1.002	1.000	0.994	1.005	1.001	0.994	1.008
Percentage of foreign prisoners	0.975	0.924	1.030	0.978	0.915	1.046	0.873	0.696	1.097
Number of years since capital punishment was abolished	0.982	0.895	1.076	0.990	0.903	1.086	1.005	0.893	1.132
Prison occupancy level	**1.059**	**1.027**	**1.093**	**1.064**	**1.029**	**1.100**	1.042	0.980	1.108
Epidemic security index (z score)	0.806	0.264	2.464	0.785	0.242	2.547	0.699	0.168	2.900
Percentage of foreign prisoners x Prison occupancy level							1.001	0.998	1.004
Control variables									
GDP per capita (ln)	4.112	0.330	51.277	3.382	0.160	71.25	2.838	0.105	76.59
Democracy (z score)	0.253	0.050	1.281	0.243	0.051	1.153	0.251	0.046	1.368
Political orientation of dominant party	2.030	0.311	13.242	1.616	0.279	9.359	1.580	0.288	8.677
Population size (ln)	1.103	0.615	1.978	0.908	0.504	1.636	0.929	0.514	1.679
Number of countries		43			41			41	
Number of adoptions		43			41			41	
Time at risk		1903			860			860	

[a] Countries in models Albania, Armenia, Austria, Azerbaijan, Belarus, Belgium, Bosnia and Herzegovina, Bulgaria, Croatia, Cyprus, Czech Republic, Denmark, Estonia, Finland, France, Georgia, Germany, Greece, Hungary, Iceland, Ireland, Italy, Kazakhstan, Latvia, Lithuania, Luxembourg, Malta, Montenegro, Netherlands, Norway Poland, Portugal, Romania, Russian Federation, Scotland, Serbia, Slovakia, Slovenia, Spain, Sweden, Switzerland, Turkey, Ukraine and United Kingdom. [b] Same countries with the exception of Slovakia and Montenegro, since these countries limited visits before the first COVD-19 case was reported. HR: Hazard Ratio. Bold numbers indicate $p < 0.05$.

Table A4.2 Survival models predicting 'Date of first early releases or pardons during pandemic at the national level.'(Exponential models)

Outcome	**Date of first early releases or pardons during pandemic at the national level**								
Onset	*January 31st, 2020-WHO declares global health emergency* [a]			*Respective date a country reports its first case of COVID-19*					
Determinants	HR	95% CI		HR	95% CI		HR	95% CI	
Prison population rate	0.997	0.992	1.002	1.000	0.994	1.005	1.00	0.99	1.01
Percentage of foreign prisoners	0.975	0.924	1.029	0.978	0.911	1.050	0.86	0.68	1.08
Number of years since capital punishment was abolished	0.982	0.896	1.075	0.989	0.890	1.098	1.01	0.89	1.15
Prison occupancy level	**1.059**	**1.033**	**1.085**	**1.071**	**1.037**	**1.107**	1.04	0.98	1.11
Epidemic security index (z score)	0.806	0.266	2.440	0.773	0.216	2.767	0.67	0.15	3.07
Percentage of foreign prisoners x Prison occupancy level							1.00	1.00	1.00
Control variables									
GDP per capita (ln)	4.076	0.327	50.833	3.897	0.153	99.10	3.00	0.09	100.6
Democracy (z score)	0.255	0.052	1.257	0.212	0.037	1.217	0.23	0.03	1.49
Political orientation of dominant party	2.013	0.337	12.034	1.875	0.276	12.74	1.78	0.31	10.24
Population size (ln)	1.099	0.630	1.918	0.976	0.540	1.762	0.98	0.56	1.74
Number of countries		43			41			41	
Number of adoptions		43			41			41	
Time at risk		1903			860			860	

[a] Countries in models Albania, Armenia, Austria, Azerbaijan, Belarus, Belgium, Bosnia and Herzegovina, Bulgaria, Croatia, Cyprus, Czech Republic, Denmark, Estonia, Finland, France, Georgia, Germany, Greece, Hungary, Iceland, Ireland, Italy, Kazakhstan, Latvia, Lithuania, Luxembourg, Malta, Montenegro, Netherlands, Norway Poland, Portugal, Romania, Russian Federation, Scotland, Serbia, Slovakia, Slovenia, Spain, Sweden, Switzerland, Turkey, Ukraine and United Kingdom. HR: Hazard Ratio. Bold numbers indicate $p < 0.05$.

Table A4·2 Different models predicting 'of first early releases or pardons during pandemic at the national level'

Outcome	**Date of first early releases or pardons during pandemic at the national level**[a]								
Models	*Logistic*			*Poisson*			*Negative binomial*		
Determinants	HR	95% CI		HR	95% CI		HR	95% CI	
Epidemic security index (z score)	0.637	0.190	2.127	0.789	0.372	1.672	0.789	0.372	1.672
GDP per capita (ln)	6.475	0.617	67.97	3.063	0.567	16.54	3.063	0.567	16.54
Population size (ln)	1.065	0.482	2.352	0.993	0.693	1.422	0.993	0.693	1.422
Democracy (z score)	0.195	0.029	1.315	0.339	0.092	1.244	0.339	0.092	1.244
Political orientation of dominant party	2.043	0.227	18.41	1.597	0.501	5.095	1.597	0.501	5.095
Prison population rate	0.998	0.987	1.008	0.997	0.992	1.002	0.997	0.992	1.002
Prison occupancy level	**1.079**	**1.036**	**1.123**	**1.040**	**1.021**	**1.060**	**1.040**	**1.021**	**1.060**
Percentage of foreign prisoners	0.961	0.918	1.005	0.976	0.943	1.010	0.976	0.943	1.010
Number of years since capital punishment was abolished	0.973	0.871	1.086	0.986	0.933	1.041	0.986	0.933	1.041
Number of countries		43			43			43	
Number of adoptions		14			14			14	

[a] Countries in models Albania, Armenia, Austria, Azerbaijan, Belarus, Belgium, Bosnia and Herzegovina, Bulgaria, Croatia, Cyprus, Czech Republic, Denmark, Estonia, Finland, France, Georgia, Germany, Greece, Hungary, Iceland, Ireland, Italy, Kazakhstan, Latvia, Lithuania, Luxembourg, Malta, Montenegro, Netherlands, Norway Poland, Portugal, Romania, Russian Federation, Scotland, Serbia, Slovakia, Slovenia, Spain, Sweden, Switzerland, Turkey, Ukraine and United Kingdom.. HR: Hazard Ratio. Bold numbers indicate $p < 0.05$.

Appendix 6

Table A6·1 Survival models predicting ‘Date of first report on implementation of visitation rights limits at the national level·’ (Weibull models excluding Belarus and Kazakhstan)

Outcome	**Date of first report on implementation of visitation rights limits at the national level**								
Onset	*January 31st, 2020-WHO declares global health emergency* [a]			*Respective date a country reports its first case of COVID-19* [b]					
Determinants	HR	95% CI		HR	95% CI		HR	95% CI	
Prison population rate	1.003	0.985	1.022	1.002	0.984	1.020	1.007	0.984	1.031
Percentage of foreign prisoners	1.004	0.982	1.025	1.004	0.982	1.026	**0.964**	**0.929**	**1.000**
Number of years since capital punishment was abolished	0.987	0.968	1.007	0.990	0.968	1.013	0.868	0.771	0.978
Prison occupancy level	1.019	0.964	1.077	1.013	0.953	1.077	**1.034**	**0.966**	**1.107**
Epidemic security index (z score)	**1.476**	**1.036**	**2.103**	**1.451**	**1.002**	**2.102**	1.347	0.929	1.954
Percentage of foreign prisoners x Prison occupancy level							**1.002**	**1.000**	**1.003**
Control variables									
GDP per capita (ln)	0.674	0.212	2.142	0.662	0.213	2.054	0.752	0.343	1.650
Democracy (z score)	1.535	0.256	9.196	1.482	0.273	8.038	1.504	0.455	4.970
Political orientation of dominant party	**3.109**	**1.812**	**5.336**	**3.081**	**1.751**	**5.421**	**3.049**	**1.768**	**5.260**
Population size (ln)	1.179	0.909	1.529	1.153	0.812	1.638	1.162	0.859	1.573
Number of countries		41			40			41	
Number of adoptions		41			40			41	
Time at risk		1858			856			860	

[a] Countries in models Albania, Armenia, Austria, Azerbaijan, Belgium, Bosnia and Herzegovina, Bulgaria, Croatia, Cyprus, Czech Republic, Denmark, Estonia, Finland, France, Georgia, Germany, Greece, Hungary, Iceland, Ireland, Italy, Latvia, Lithuania, Luxembourg, Malta, Montenegro, Netherlands, Norway Poland, Portugal, Romania, Russian Federation, Scotland, Serbia, Slovakia, Slovenia, Spain, Sweden, Switzerland, Turkey, Ukraine and United Kingdom.. [b] Same countries except for Slovakia and Montenegro, since these countries limited visits before the first COVD-19 case was reported. HR: Hazard Ratio. Bold numbers indicate $p < 0.05$.

Table A5·2 Different models predicting 'Date of first report on implementation of visitation rights limits at the national level.'(Excluding Belarus and Kazakhstan)

Outcome	**Date of first early releases or pardons during pandemic at the national level[a]**								
Models		*Logistic*			*Poisson*			*Negative binomial*	
Determinants	HR	95% CI		HR	95% CI		HR	95% CI	
Epidemic security index (z score)	0.700	0.180	2.725	0.83	0.38	1.78	0.825	0.384	1.776
GDP per capita (ln)	9.303	0.368	235.3	4.04	0.50	32.59	4.041	0.501	32.58
Population size (ln)	1.101	0.490	2.473	1.01	0.71	1.44	1.013	0.712	1.442
Democracy (z score)	0.113	0.008	1.542	0.24	0.05	1.10	0.237	0.051	1.100
Political orientation of dominant party	2.159	0.225	20.72	1.58	0.52	4.81	1.576	0.517	4.808
Prison population rate	0.994	0.972	1.015	0.99	0.99	1.00	0.995	0.986	1.004
Prison occupancy level	**1.071**	**1.032**	**1.111**	**1.04**	**1.02**	**1.06**	**1.037**	**1.017**	**1.056**
Percentage of foreign prisoners	0.957	0.908	1.009	0.97	0.93	1.01	0.973	0.934	1.013
Number of years since capital punishment was abolished	0.966	0.852	1.095	0.98	0.93	1.04	0.983	0.926	1.043
Number of countries		41			41			41	
Number of adoptions		14			14			14	

[a] Countries in models Albania, Armenia, Austria, Azerbaijan, Belgium, Bosnia and Herzegovina, Bulgaria, Croatia, Cyprus, Czech Republic, Denmark, Estonia, Finland, France, Georgia, Germany, Greece, Hungary, Iceland, Ireland, Italy, Latvia, Lithuania, Luxembourg, Malta, Montenegro, Netherlands, Norway Poland, Portugal, Romania, Russian Federation, Scotland, Serbia, Slovakia, Slovenia, Spain, Sweden, Switzerland, Turkey, Ukraine and United Kingdom.. HR: Hazard Ratio. Bold numbers indicate $p < 0.05$.

Appendix 6

Table A6.1 Survival models predicting 'Date of first report on implementation of visitation rights limits at the national level.' with capital punishment as dichotomous variable (Weibull models).

Outcome	**Date of first report on implementation of visitation rights limits at the national level[a]**								
Onset	*January 31st, 2020-WHO declares global health emergency [a]*			*Respective date a country reports its first case of COVID-19 [b]*					
Determinants	HR	95% CI		HR	95% CI		HR	95% CI	
Prison population rate	1.003	0.991	1.015	1.001	0.989	1.013	1.006	0.989	1.022
Percentage of foreign prisoners	1.005	0.987	1.023	1.004	0.985	1.024	**0.872**	**0.796**	**0.955**
Capital punishment abolished	1.019	0.976	1.063	1.011	0.960	1.064	1.030	0.975	1.087
Prison occupancy level	0.987	0.967	1.007	0.990	0.967	1.012	**0.964**	**0.933**	**0.996**
Epidemic security index (z score)	**1.507**	**1.061**	**2.142**	**1.476**	**1.029**	**2.118**	1.391	0.966	2.004
Percentage of foreign prisoners x Prison occupancy level							**1.001**	**1.000**	**1.003**
Control variables									
GDP per capita (ln)	0.683	0.335	1.391	0.703	0.339	1.459	0.773	0.303	1.975
Democracy (z score)	1.527	0.552	4.228	1.346	0.474	3.820	1.143	0.814	1.604
Political orientation of dominant party	**3.200**	**1.843**	**5.556**	**3.074**	**1.717**	**5.501**	1.359	0.346	5.346
Population size (ln)	1.202	0.928	1.556	1.164	0.824	1.643	**2.810**	**1.531**	**5.160**
Number of countries		43			41			41	
Number of adoptions		43			41			41	
Time at risk		1903			860			860	

[a] Countries in models Albania, Armenia, Austria, Azerbaijan, Belarus, Belgium, Bosnia and Herzegovina, Bulgaria, Croatia, Cyprus, Czech Republic, Denmark, Estonia, Finland, France, Georgia, Germany, Greece, Hungary, Iceland, Ireland, Italy, Kazakhstan, Latvia, Lithuania, Luxembourg, Malta, Montenegro, Netherlands, Norway Poland, Portugal, Romania, Russian Federation, Scotland, Serbia, Slovakia, Slovenia, Spain, Sweden, Switzerland, Turkey, Ukraine, and United Kingdom. [b] Same countries except for Slovakia and Montenegro, since these countries limited visits before the first COVD-19 case was reported.HR: Hazard Ratio. Bold numbers indicate $p < 0.05$.

Table A6.2 Survival models predicting 'Date of first early releases or pardons during pandemic at the national level' with capital punishment as dichotomous variable (Weibull models).

Outcome	**Date of first early releases or pardons during pandemic at the national level[a]**								
Onset	*January 31st, 2020-WHO declares global health emergency [a]*			*Respective date a country reports its first case of COVID-19*					
Determinants	HR	95% CI		HR	95% CI		HR	95% CI	
Prison population rate	0.997	0.989	1.004	1.000	0.993	1.007	1.000	0.992	1.007
Percentage of foreign prisoners	0.980	0.941	1.021	0.980	0.936	1.026	0.980	0.937	1.025
Capital punishment abolished	2.019	0.244	16.703	2.734	0.449	16.63	2.832	0.579	13.85
Prison occupancy level	**1.061**	**1.010**	**1.114**	**1.063**	**1.014**	**1.113**	**1.062**	**1.014**	**1.112**
Epidemic security index (z score)	0.742	0.297	1.856	0.721	0.287	1.810	0.718	0.281	1.834
Percentage of foreign prisoners x Prison occupancy level							1.001	0.982	1.020
Control variables									
GDP per capita (ln)	4.933	0.323	75.341	4.791	0.245	93.70	4.828	0.273	85.42 3
Democracy (z score)	1.142	0.586	2.227	0.961	0.471	1.960	0.960	0.475	1.938
Political orientation of dominant party	0.126	0.014	1.108	0.124	0.012	1.247	0.123	0.014	1.048
Population size (ln)	2.168	0.210	22.420	1.843	0.215	15.78	1.830	0.227	14.75 0
Number of countries		43			43			43	
Number of adoptions		14			14			14	
Time at risk		5336			4288			4288	

[a] Countries in models Albania, Armenia, Austria, Azerbaijan, Belarus, Belgium, Bosnia and Herzegovina, Bulgaria, Croatia, Cyprus, Czech Republic, Denmark, Estonia, Finland, France, Georgia, Germany, Greece, Hungary, Iceland, Ireland, Italy, Kazakhstan, Latvia, Lithuania, Luxembourg, Malta, Montenegro, Netherlands, Norway Poland, Portugal, Romania, Russian Federation, Scotland, Serbia, Slovakia, Slovenia, Spain, Sweden, Switzerland, Turkey, Ukraine, and United Kingdom.·. HR: Hazard Ratio. Bold numbers indicate $p < 0.05$.
